# Genomic identification and expression analysis of nuclear pore proteins in *Malus domestica*

**DOI:** 10.1038/s41598-020-74171-0

**Published:** 2020-10-15

**Authors:** Chenguang Zhang, Na An, Peng Jia, Wei Zhang, Jiayan Liang, Xu Zhang, Hua Zhou, Wenchun Ma, Mingyu Han, Libo Xing, Xiaolin Ren

**Affiliations:** College of Horticulture, Northwest A&F University, Yangling, China

**Keywords:** Developmental biology, Genetics, Plant sciences

## Abstract

The nuclear pore complex (NPC), comprised of individual nucleoporin (Nup) proteins, controls nucleo-cytoplasmic transport of RNA and protein, and is important for regulating plant growth and development. However, there are no reports on this complex in fruit tree species. In this study, we identified 38 apple Nups and named them based on the known *Arabidopsis thaliana* homologs. We also completed bioinformatics analyses of the intron and exon structural data for apple Nups. The proteins encoded by the apple Nups lacked a universally conserved domain. Moreover, a phylogenetic analysis separated the apple and *A. thaliana* Nups into three groups. The phylogenetic tree indicated that *MdNup54* and *MdNup62* are most closely related to genes in other Rosaceae species. To characterize the 38 candidate *Malus domestica* Nups, we measured their stage-specific expression levels. Our tests revealed these proteins were differentially expressed among diverse tissues. We analyzed the expression levels of seven apple Nups in response to an indole-3-acetic acid (IAA) treatment. The phytohormone treatment significantly inhibited apple flowering. A qRT-PCR analysis proved that an IAA treatment significantly inhibited the expression of these seven genes. A preliminary study regarding two members of the *Nup62* subcomplex, *MdNup54* and *MdNup62*, confirmed these two proteins can interact with each other. A yeast two-hybrid assay verified that *MdNup54* can interact with *MdKNAT4* and *MdKNAT6*. On the basis of the study results, we identified apple NPC and predicted its structure and function. The data generated in this investigation provide important reference material for follow-up research.

## Introduction

The nuclear pore complex (NPC), located within invaginations of the nuclear envelope, is a massive macromolecular conglomerate in cells^1^. It is composed of multiple copies of at least 30 diverse nucleoporins (Nups)^2^. Materials transported between the nucleus and cytoplasm have important effects on cell functions^3^, and the NPC is the only channel that controls nucleo-cytoplasmic transport of RNA and protein^3,4^. The initial research regarding the NPC involved analyses of vertebrates and yeast, and revealed that the Nups of yeast and metazoans are approximately 60 and 120 MDa, respectively^1^. An ultrastructural analysis indicated that the basic NPC framework is conserved in vertebrates, yeast, and plants^2,5–7^. The following three subcomplexes have been identified in vertebrate NPC: the *Nup107* subcomplex (*Nup37, Nup43, Nup85, Nup96, Nup107, Nup133,Nup160, Seh1,* and *Sec13*); the *Nup62* subcomplex (*Nup45 , Nup54, Nup58,* and *Nup62*); and the *Nup93* subcomplex (*Nup35, Nup93,Nup155, Nup188,* and *Nup205*)^8^. The different subcomplexes have diverse functions. The remaining members *Nup50*, *Nup88*, *Nup98*, *Nup136*, *NDC1*, *Tpr/NUA*, *CG1*, *RAE1a/b*, *ALADIN*, *GP210*, *HOS1*, , and *GLE1* do not form a subcomplex^2^, and they are also important parts of the NPC. Relatively little was known about plant NPC until recent studies involving electron microscopy^7^, proteomics^2^, and bioinformatics analyses confirmed that the NPCs of plants and other eukaryotes are structurally similar^9,10^. Tamura uncovered a greater sequence homology between plant and vertebrate NPCs than between plant and yeast NPCs^2^.

The research to date on plant NPC has revealed it influence plant immunity^11–13^, hormone signaling^14–18^, abiotic stress responses^2,19–21^, and flowering^1–3,22,23^. The *mos3 Arabidopsis thaliana* deletion mutant, which lacks a homolog of the *Nup96* gene in animals, is more susceptible to pathogens than normal^11^. Additionally, *MOS7*, *Seh1*, and *Nup160* are also involved in the plant immune pathway^12,13,17^. *Nup160* and *Nup96* mutations affect the nuclear output of mRNA and the subcellular localization of the auxin response transcriptional repressor IAA17 protein in *A. thaliana*, thereby partially restoring the auxin-resistant phenotype of *axr-1* mutants^14^. Thus, Nup160 and Nup96 are likely involved in the auxin signal transduction pathway. The *tpr/mlp1p/mlp2p* mutations result in phenotypes that are similar to those due to mutations to *Nup160* and *Nup96*^15,16^. In addition to altering auxin signal transduction, a mutation to *Nup160* also increases the responsiveness of *A. thaliana* to ethylene, suggesting that it may help mediate the interaction between auxin and ethylene signals^18^. In terms of abiotic stress responses, both *Nup160* and *HOS1* participated in chilling stress by regulating CBF gene^19–21^. Furthermore, The HOS1 protein can specifically mediate the degradation of the ICE1 protein under cold conditions, thereby weakening *A. thaliana* responses to low temperatures^21^.And the *Nup85* mutant reduced ABA and salt stress response in *A. thaliana*^22^. Additionally, *Nup160*, *Nup96*, and *HOS1* also affect the flowering time of plants^3^. Specifically, *HOS1* interacts with some nuclear genes to regulate its binding to *FLC* chromatin in flowering plants at low temperatures and weaken the transcriptional inhibition of *FLC* by *HDA6*^23^. In *A. thaliana*, *HOS1* interacts directly with *CO*. In the *hos1* mutant, *CO* accumulates, which inhibits *FLC* expression and ultimately promotes flowering^24^. Mutations to *Nup54*, *Nup58*, *Nup62*, *Nup136*, and *Nup160* result in an obvious early flowering phenotype in *A. thaliana*, whereas mutations to *Nup62-2* and *Nup160-4* lead to dwarfism^1,2^. An investigation of *A. thaliana* proved that *Nup96* promotes the stability of *HOS1*, which binds to and degrades *CO*, resulting in delayed flowering. Moreover, *HOS1* increases the stability of *Nup96*, thereby maintaining this regulatory pathway to control flowering time^25^.

A lot of research has been done on the model plants, which gives us a certain understanding of the plant NPC^3,4,26^. However, there is no report on NPC research of woody fruit trees. Considering the important function of NPC, it is necessary to carry out the related research on woody fruit trees. Apple is one of the most important fruit tree species worldwide. So we first identified candidate apple Nups based on *A. thaliana* Nups, after which we characterized the gene structure, protein structure, and tissue-specific expression patterns. We know that most apple species produce relatively few flowers or have stunted flower buds, which seriously affects the apple industry^27,28^. Although previous research has confirmed that some Nups in *A. thaliana* are involved in the flowering pathway, there have been no similar studies of apple Nups. So we made statistics on the effect of IAA treatment on apple flowering rate and detected the expression of some Nups to preliminarily reveal the effect of apple Nups on flowering. In addition, we conducted a preliminary study on the Nup62 subcomplex of apple. We studied the interaction between *MdNup62* and *MdNup54* and screened for proteins that interact with *MdNup54*. To the best of our knowledge, this study is the first comprehensive survey of the apple NPC, and the data presented herein will be useful for future analyses.

## Materials and methods

### Plant materials and treatments

The roots, stems, leaves, buds, flowers, and fruits of 6-year-old apple trees (Fu ji/T337/*Malus robusta Rehd*.) were collected for a tissue-specific gene expression analysis. We collected newly grown lateral roots (1–2 mm in diameter), new shoots (2–3 mm in diameter) near the tip, fully expanded leaves near buds, flower buds, blooming flowers, and young fruits, which were immediately frozen in liquid nitrogen and stored at − 80 °C for later use.

Regarding the hormone treatment, 40 apple trees (108° 04′ E, 34° 16′ N) growing in the experimental orchard of the Horticulture College of Northwest A&F University were randomly divided into two groups, which were treated with 300 mg/L IAA or water. During the study, apple leaves were dusted with a low-pressure manual duster, and samples were collected at 30, 50, and 70 days after flowering. The samples were immediately frozen in liquid nitrogen and stored at − 80 °C.

We also investigated the effects of an IAA treatment on the flowering rate of apple trees. Specifically, five similarly growing IAA- and water-treated apple trees were examined. The flowering rate was calculated as previously described^29^.

### Identification of apple NPC

To identify apple NPC, we used the 30 identified NPC protein sequences of *A. thaliana* as queries to search the apple genome database (*Malus domestica* Genome GDDH13 V1.1, https://www.rosaceae.org/). The obtained sequences were then used as queries to search the conserved domain database (https://www.ncbi.nlm.nih.gov/Structure/cdd/wrpsb.cgi). The genes lacking the relevant Nup domain were eliminated. All non-redundant putative protein sequences were finally manually checked to confirm the presence of the Nup domain.

### Analyses of phylogenetic relationships, gene structures, and tertiary protein structures

A phylogenetic tree comprising apple and *A. thaliana* Nups as well as *Nup54* and *Nup62* from 10 species (*Arabidopsis thaliana*, *Malus domesica*, *Populus trichocarpa*, *Oryza sativa*, *Rosa chinensis*, *Pyrus communis*, *Ananas comosus*, *Vitis vinifera*, *Zea mays* , and *Prunus persica*) were constructed with the MEGA-X program. The Gene Structure Display Server (https://gsds.cbi.pku.edu.cn/) was used to construct exon–intron structures. The gene structures were determined based on the coding sequences within the corresponding genomic sequences. The predicted Apple NPC tertiary structures were analyzed with the PHYRE server (version 2.0) (https://www.sbg.bio.ic.ac.uk/phyre2/html/page.cgi?id=index).

### RNA extraction and qRT-PCR analysis

Total RNA was extracted from apple buds with the RNA Plant Plus Reagent Kit (TIANGEN, Beijing, China). The RNA was used as the template to synthesize cDNA with the PrimeScript RT Reagent Kit (TAKARA, Shiga, Japan). The expression levels of all identified Apple Nups were analyzed by qRT-PCR with primer pairs designed with Primer 6.0 (Table S1). The qRT-PCR analysis was conducted with the StepOnePlus Real-Time PCR System (THERMO FISHER SCIENTIFIC, USA). The reaction solution comprised 10 μL SYBR Green I Master Mix (CWBIO, Beijing, China), 0.5 μmol L^−1^ primers (SANGON BIOTECH, Shanghai, China), and 1 μL each template in a total volume of 20 μL.

The PCR program was as follows: 95 °C for 3 min; 40 cycles of 94 °C for 15 s, 62 °C for 20 s, and 72 °C for 20 s. The resulting fragments were immediately subjected to a melting-curve analysis to verify the amplification of gene-specific PCR products. The melting-curve analysis was completed with the following program: 94 °C for 15 s, followed by a constant increase from 60 to 95 °C at a 2% ramping rate. The apple actin gene (MD04G1127400) was used as an internal standard. All samples were analyzed with three biological replicates, each comprising three technical replicates. Relative gene expression levels were calculated according to the 2^−ΔΔCt^ method^30^.

### Yeast two-hybrid (Y2H) assay

The *MdNup54*^175–339^ and *MdNup62*^508–613^ truncated sequences were cloned into the pGBKT7 vector to generate the *MdNup54*^175–339^-pGBKT7 and *MdNup62*^508–613^-pGBKT7 recombinant plasmids. The *MdNup54*, *MdKNAT4*, and *MdKNAT6* open reading frames were inserted into the pGADT7 vector to generate the *MdNup54*-pGADT7, *MdKNAT4*-pGADT7, and *MdKNAT6*-pGADT7 recombinant plasmids. The recombinant plasmids were inserted into Gold Yeast Two-Hybrid cells, which were then grown on selective medium. The primers used in the yeast experiment were designed with Primer 6.0 (Table S2). And these four gene sequences were submitted to NCBI (*MdNup54*: MT102239, *MdNup62*: MT102240, *MdKNAT4*: MT102238, and *MdKNAT6*: MT102237).

### Split luciferase (LUC) complementation

The full-length *MdNup54* coding sequence was cloned into CLUC vectors, whereas *MdNup62* was cloned into NLUC vectors. The split-LUC complementation assay was performed with tobacco leaves. The LUC activity was quantified with the Dual-Luciferase Reporter Assay System. The primers used in the luciferase experiment were designed with Primer 6.0 (Table S2).

### Statistical analysis

Data underwent an analysis of variance and the means were compared with a *t*-test at the 5% level using the SPSS 11.5 software package. Figures were prepared with Excel.

## Results

### Genome-wide identification of NPC in apple

**Figure 1:**
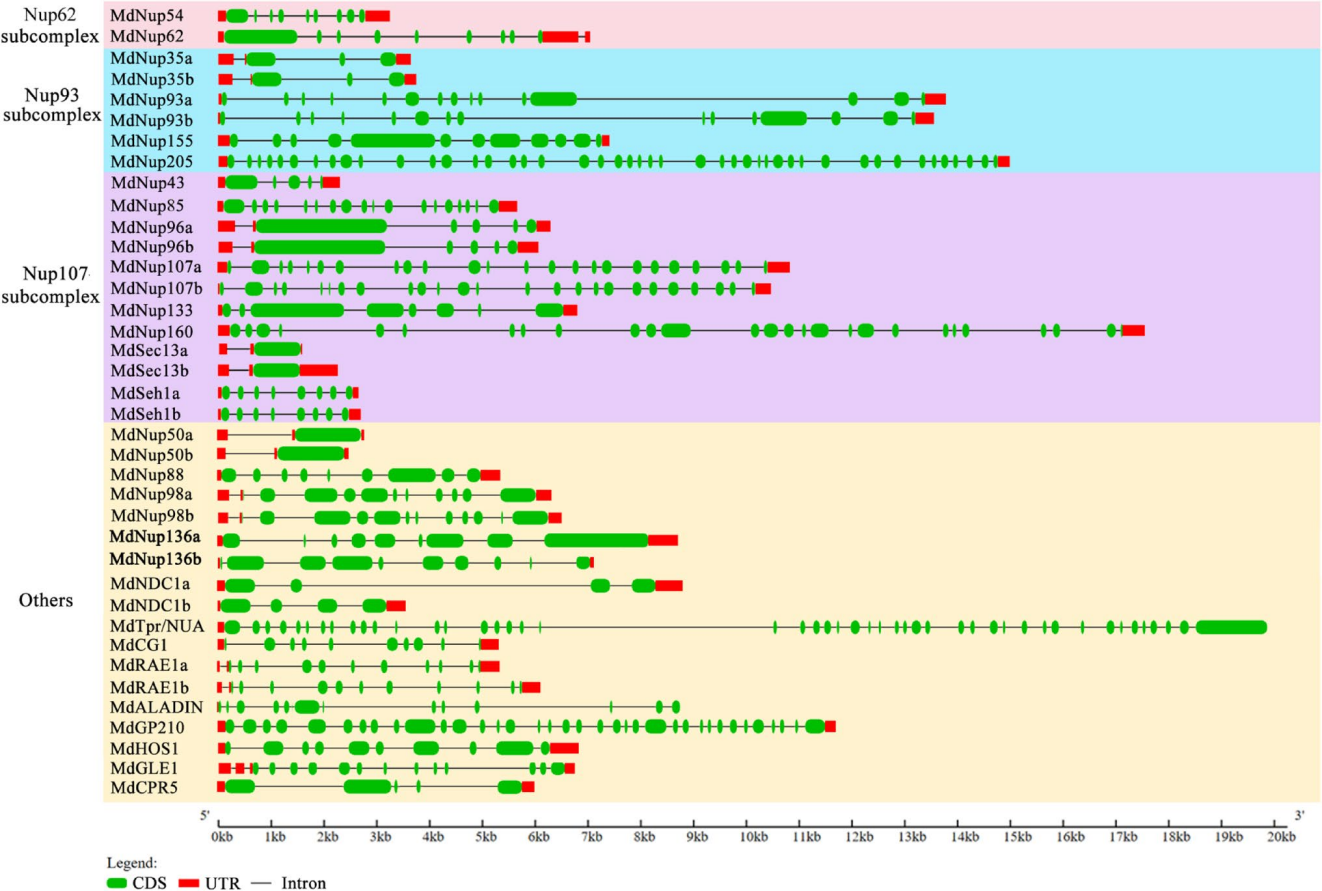
Analysis of apple NPC gene structures. The Gene Structure Display Server (https://gsds.cbi.pku.edu.cn/) was used to construct exon–intron structures. Green boxes and black lines refer to exons and introns, respectively. Red boxes represent untranslated regions.

The apple Nups were detected and identified in the GDR database using BlastP. We obtained 38 candidate apple Nups after the genes with incomplete Nup-related domains and recurring genes were eliminated. The identified apple Nups are *MdNup35a/b*, *MdNup43*, *MdNup50a/b*, *MdNup54*, *MdNup62*, *MdNup85*, *MdNup88*, *MdNup93a/b*, *MdNup96a/b*, *MdNup98a/b*, *MdNup107a/b*, *MdNup133*, *MdNup136a/b*, *MdNup155*, *MdNup160*, *MdNup205, MdSec13a/b*, *MdSeh1a/b*, *MdNDC1a/b*, *MdTpr/NUA*, *MdCG1*, *MdRAE1a/b*, *MdALADIN*, *MdGP210*, *MdHOS1*, *MdGLE1*, and *MdCPR5* (Table 1). Figure 1 also shows the gene locus, location, sequence length and other information of apple NUPs.

**Table 1:** Information on the Nups in apple.

Subcomplex	Gene	Gene locus	Location	CDS (bp)	Peptide (aa)	AtNPC	E value
Nup62 subcomplex	MdNup54	MD16G1117500	Chr16:8338184..8341499	1200	400	AtNup54	1.5394E−161
	MdNup62	MD07G1110700	Chr07:12755345..12762516	2172	724	AtNup62	1.69142E−135
Nup93 subcomplex	MdNup35a	MD09G1205800	Chr09:19658131..19661853	993	331	AtNup35	9.05037E−151
	MdNup35b	MD17G1186700	Chr17:22318731..22322549	993	331	AtNup35	3.02073E−147
	MdNup93a	MD12G1080600	Chr12:9823197..9837227	2592	864	AtNup93	0
	MdNup93b	MD14G1076700	Chr14:8681861..8695666	2589	863	AtNup93	0
	MdNup155	MD13G1020400	Chr13:1280351..1287904	4407	1469	AtNup155	0
	MdNup205	MD02G1032900	Chr02:2574878..2590132	5643	1881	AtNup205	0
Nup107 subcomplex	MdNup43	MD10G1281400	Chr10:37201121..37203464	1053	351	AtNup43	1.53139E−127
	MdNup85	MD15G1093200	Chr15:6453444..6459227	2175	725	AtNup85	0
	MdNup96a	MD08G1215300	Chr08:27827088..27833504	3096	1032	AtNup96	0
	MdNup96b	MD15G1399200	Chr15:50011301..50017471	3348	1116	AtNup96	0
	MdNup107a	MD09G1178100	Chr09:15235815..15246849	3246	1082	AtNup107	0
	MdNup107b	MD17G1148600	Chr17:13573595..13584264	3219	1073	AtNup107	0
	MdNup133	MD17G1113600	Chr17:9738615..9745545	3915	1305	AtNup133	0
	MdNup160	MD10G1009800	Chr10:1350846..1368727	4521	1507	AtNup160	0
	MdSec13a	MD09G1041000	Chr09:2640031..2641626	903	301	AtSeh13	1.71982E−170
	MdSec13b	MD17G1042300	Chr17:3101074..3103384	903	301	AtSeh13	4.377E−173
	MdSeh1a	MD06G1103700	Chr06:24176846..24179550	978	326	AtSeh1	2.26035E−138
	MdSeh1b	MD14G1122800	Chr14:19731969..19734722	981	327	AtSeh1	8.91422E−139
Others	MdNup50a	MD09G1214400	Chr09:21079805..21082646	1272	424	AtNup50a	4.0775E−100
	MdNup50b	MD17G1196800	Chr17:23575668..23578205	1293	431	AtNup50a	3.54614E−76
	MdNup88	MD01G1152200	Chr01:26080908..26086372	2430	810	AtNup88	0
	MdNup98a	MD06G1126300	Chr06:26827660..26834106	2973	991	AtNup98a/b	0
	MdNup98b	MD14G1142000	Chr14:23465594..23472220	3069	1023	AtNup98a/b	0
	MdNup136a	MD02G1257800	Chr02:31057936..31066675	3885	1294	AtNup136	5.85327E−125
	MdNup136b	MD07G1063100	Chr07:5916974..5924113	2997	998	AtNup136	2.51999E−117
	MdNDC1a	MD05G1278400	Chr05:41276844..41285832	1647	549	AtNDC1	1.18565E−172
	MdNDC1b	MD10G1257000	Chr10:35129858..35133484	1653	551	AtNDC1	2.80708E−159
	MdTpr/NUA	MD05G1240600	Chr05:37117000..37137248	6306	2102	AtTpr/NUA	0
	MdCGI	MD04G1000600	Chr04:62175..67597	1173	391	AtCG1	3.48934E−63
	MdRAE1a	MD08G1221600	Chr08:28411242..28416688	1044	348	AtRAE1	0
	MdRAE1b	MD15G141250	Chr15:51213459..51219697	1032	344	AtRAE1	0
	MdALADIN	MD12G1112000	Chr12:17757407..17766342	1638	546	AtALADIN	1.53421E−105
	MdGP210	MD17G1026700	Chr17:1907720..1919647	5913	1971	AtGP210	0
	MdHOS1	MD04G1060900	Chr04:8017657..8024612	2919	973	AtHOS1	0
	MdGLE1	MD13G1104500	Chr13:7472044..7478918	1935	645	AtGLE1	1.86679E−127
	MdCPR5	MD01G1017700	Chr01:7756340..7761421	1860	620	AtCPR5	2.16518E−85

### Gene structures in apple Nups

To structurally characterize the identified apple Nups, we generated exon–intron diagrams and revealed the coding sequences and untranslated regions (Fig. 1). An examination of all apple Nups indicated that *MdTpr/NUA* has the most exons, with 47, whereas *MdSec13a/b* and *MdNup50a/b* have the fewest exons, with only two. Of the Nup62 subcomplex genes, *MdNup54* and *MdNup62* have 9 and 10 exons, respectively. Regarding the Nup93 subcomplex, *MdNup205* has the most exons, with 45, and *MdNup35* has the fewest exons, with only four. The mean number of exons in the Nup93 subcomplex genes is 15.83. Among the Nup107–160 subcomplex genes, *MdNup160* has the most exons, with 27, whereas *MdSec13a/b* has the fewest exons, with two. The mean number of exons is 11.58. Of the Other analyzed genes, *MdTpr/NUA* has the most exons, with 47, and *MdNup50a/b* has the fewest exons, with 2. The mean number of exons is 13.50. Additionally, we also predicted the tertiary structures of the Apple Nups, revealing α helices, β sheets, and random coils in all proteins (Fig. S1).

### Conserved protein domains in apple Nups

**Figure 2:**
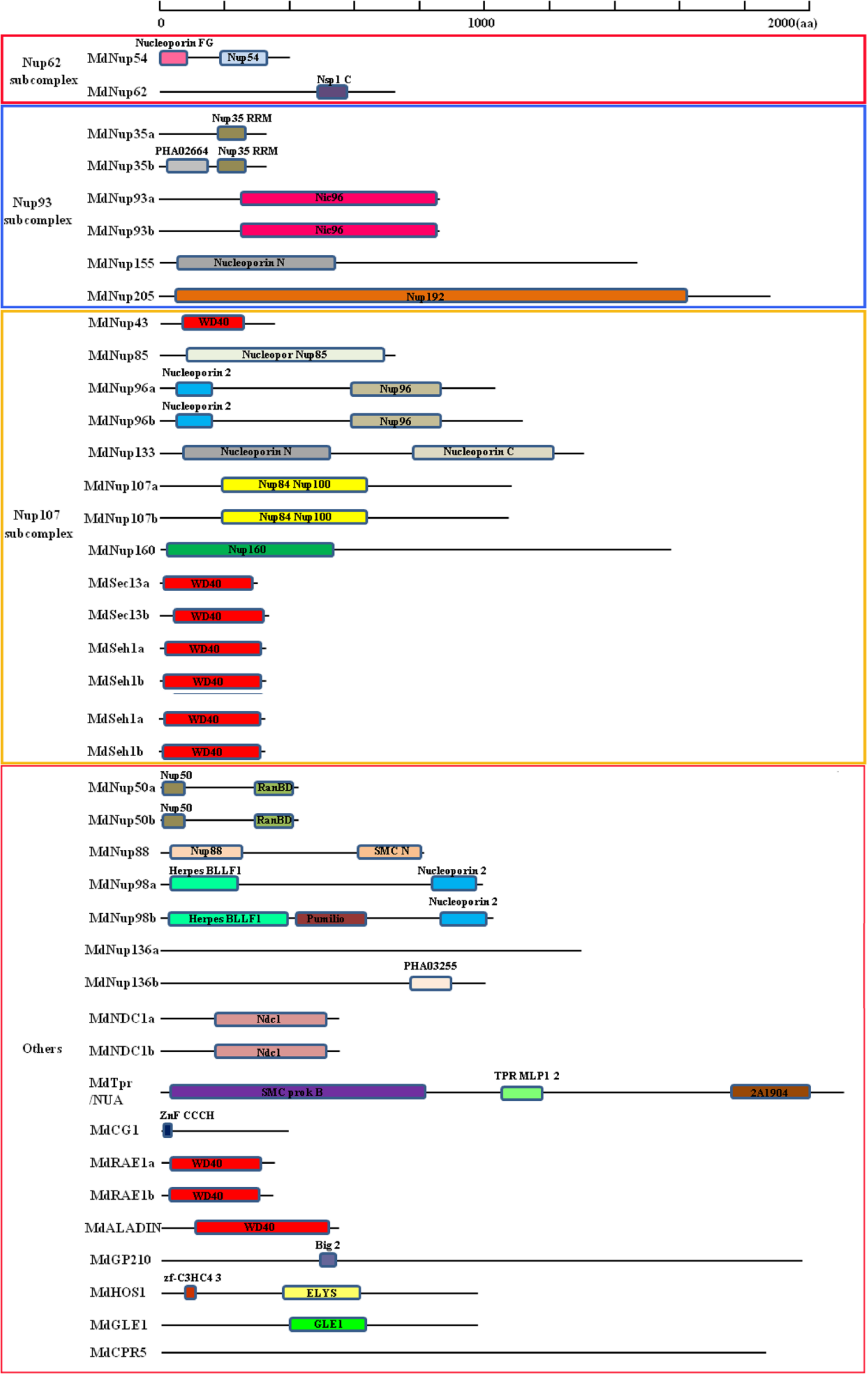
Schematic representation of the predicted domain features of apple NPCs. BlastP in NCBI (https://www.ncbi.nlm.nih.gov/Structure/cdd/wrpsb.cgi) was used to get predicted domain information. Different squares represent different conserved domains of the protein, and the black line represents the number of amino acids.

We used the NCBI BlastP tool to analyze the conserved protein domains of 38 apple Nups. As a class of complexes, Apple Nups have no common conserved domain. However, there are domains that are conserved among some members. For example, *MdNup133* and *MdNup155* have a conserved Nucleoporin N structural domain, whereas *MdNup43*, *MdSec13a/b*, *MdSeh1a/b*, *MdRAE1a/b*, and *MdALADIN* share a common conserved WD40 structural domain (Fig. 2). On the basis of previous studies, we divided the apple Nups into the following four subcomplex categories: *Nup62* subcomplex, *Nup93* subcomplex, *Nup107* subcomplex and others. And there are 2 members (*MdNup54* and *MdNup62*) in *Nup62* subcomplex, 6 members (*MdNup35a/b*, *MdNup93a/b*, *MdNup155*, and *MdNup205*) in *Nup93* subcomplex, 12 members (*MdNup43*, *MdNup85*, *MdNup96a/b*, *MdNup107a/b*, *MdNup133*, *MdNup160*, *MdSec13a/b*, and *MdSeh1a/b*) in *Nup107* subcomplex, and 18 members (*MdNup50a/b*, *MdNup88*, *MdNup98a/b*, *MdNup136a/b*, *MdNDC1a/b*, *MdTpr/NUA*, *MdCG1*, *MdRAE1a/b*, *MdALADIN*, *MdGP210*, *MdHOS1*, *MdGLE1*, and *MdCPR5*) in Others.

### Analysis of evolutionary relationships among Nups

**Figure 3:**
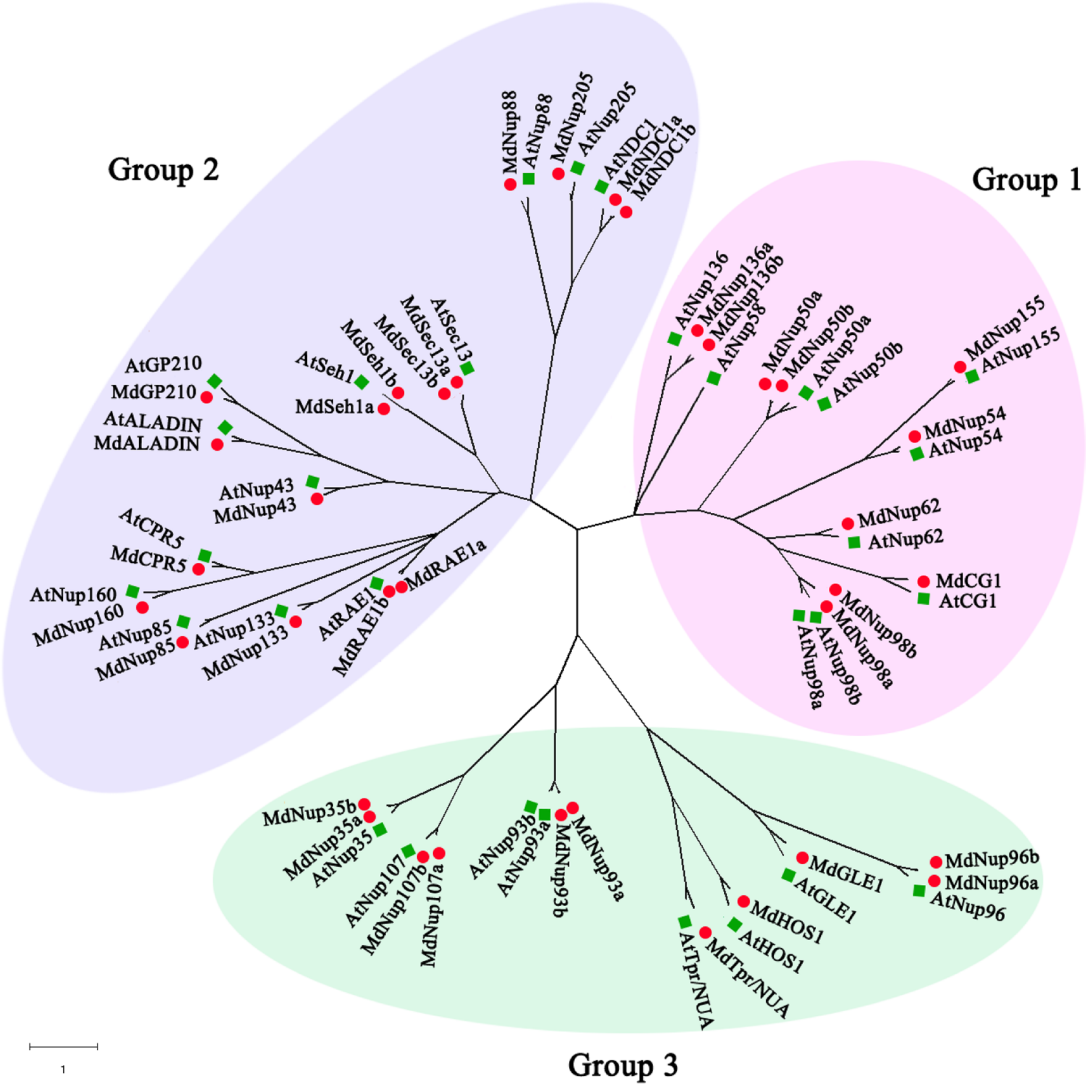
Phylogenetic analysis of apple and *Arabidopsis thaliana* NPCs. The phylogenetic trees were obtained through MEGA-X. *Arabidopsis thaliana*: At, green square; apple: Md, red circle.

To elucidate the evolutionary relationships among Nups, we constructed a phylogenetic tree consisting of *A. thaliana* and apple Nups (Fig. 3). The Nups were divided into three groups, Groups 1, 2, and 3, which comprised 20, 30, and 19 members, respectively. Group 1 had 10 apple genes (*MdNup50a/b, MdNup54, MdNup62, MdNup98a/b, MdNup136a/b*, *MdNup155,* and *MdCG1*), while 17 apple genes (*MdNup43*, *MdNup85*, MdNup88, *MdNup133*, *MdNup160*, *MdNup205, MdALADIN, MdNDC1a/b, MdGP210, MdRAE1a/b, MdSec13a/b, MdCPR5*, and *MdSeh1a/b*) were clustered in Group 2 and 11 apple genes (*MdNup35a/b*, *MdNup93a/b*, *MdNup96a/b*, *MdNup107a/b*, *MdGLE1*, *MdHOS1,* and *MdTpr/NUA*) were clustered in Group 3.

### Evolutionary relationship between *MdNup54* and *MdNup62* among plant species

**Figure 4:**
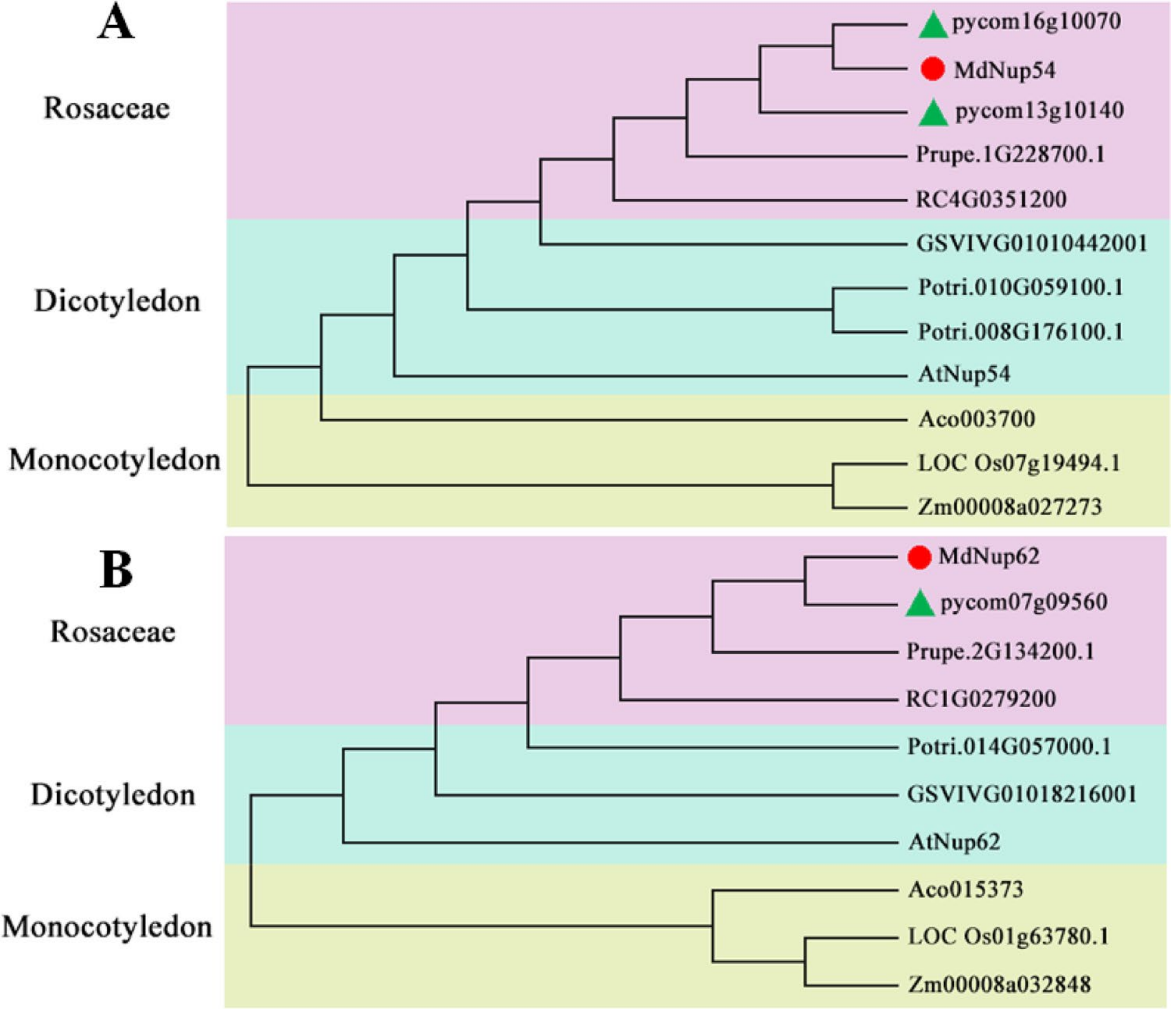
Phylogenetic analysis of *Nup54* (**a**) and *Nup62* (**b**) in 10 species (*Arabidopsis thaliana*, apple, poplar, rice, rose, pear, pineapple, grape, corn, and peach). The phylogenetic trees were obtained through MEGA-X. The red circle represents apple gene, and the green triangle represents pear gene.

*Nup62* subcomplex is located in the central part of the nuclear pore and plays an important role in the regulation of substances into and out of the nucleus^1,2^. And it has only two members in apple, *MdNup54* and *MdNup62*. Given their importance, we analyzed the evolutionary relationship between *Nup54* and *Nup62* among the 10 species (*Arabidopsis thaliana*, *Malus domestica*, *Populus trichocarpa*, *Oryza sativa*, *Rosa chinensis*, *Pyrus communis*, *Ananas comosus*, *Vitis vinifera*, *Zea mays* , and *Prunus persica*) (Fig. 4). A phylogenetic analysis indicated that both *MdNup54* and *MdNup62* are closely related to genes in *Rosa chinensis*, *Pyrus communis*, and *Prunus persica* in the family Rosaceae, but are more distantly related to genes in monocotyledons (*Oryza sativa*, *Ananas comosus*, and *Zea mays*).

### Expression levels of apple Nups in various tissues

**Figure 5:**
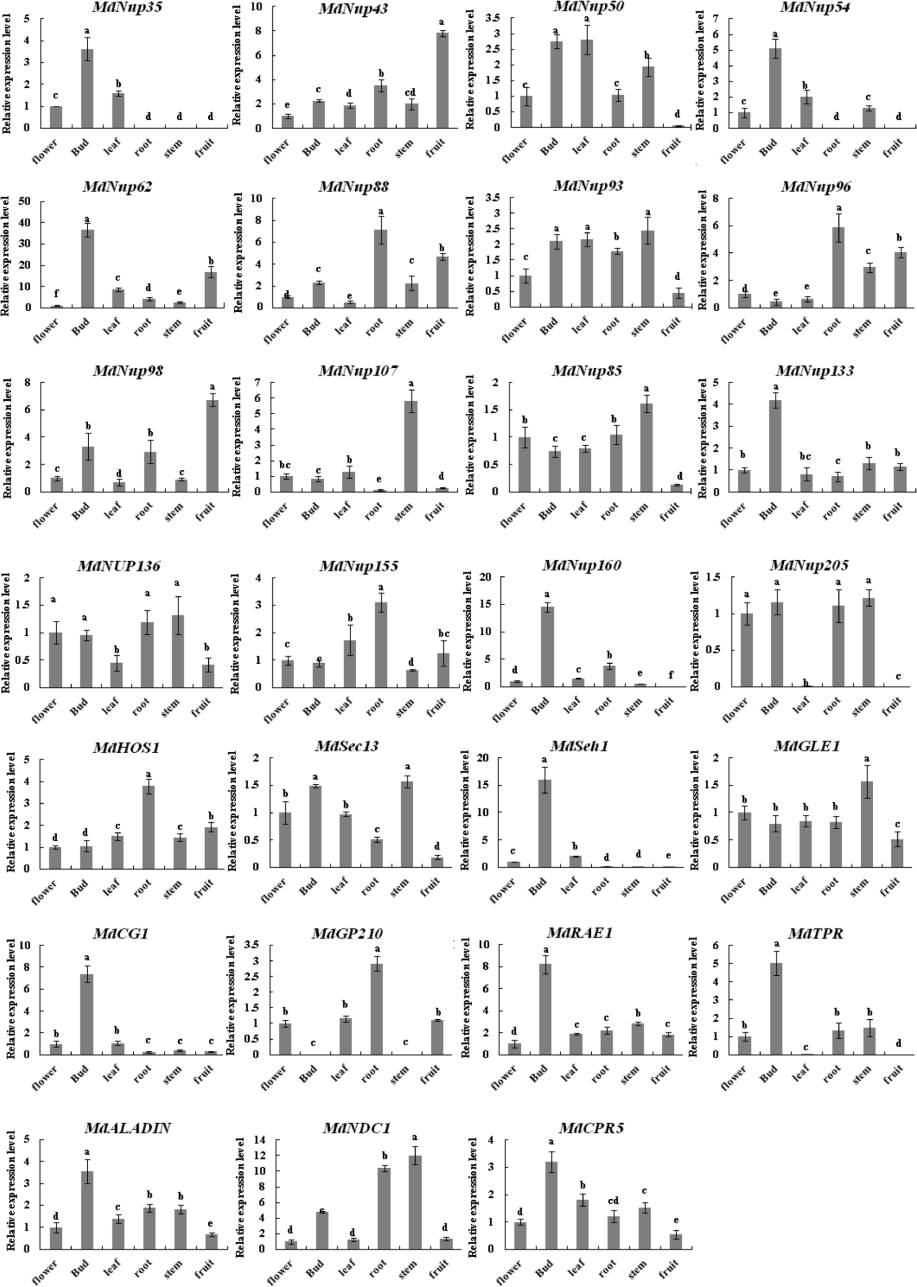
Analysis of *MdNPC* expression levels in diverse ‘Nagafu No. 2’ tissues. Each sample was analyzed with three biological replicates, each comprising three technical replicates. The histograms were made by Excel 2007. Means followed by different lowercase letters are significantly different at the 0.05 level.

To functionally characterize apple Nups in apple, we completed a qRT-PCR assay to determine apple Nups expression levels in diverse tissues (flowers, buds, leaves, roots, stems and fruits) (Fig. 5). Because there are 11 pairs of highly similar homologous apple Nups (*MdNup35a/b*, *MdNup93a/b*, *MdNup96a/b*, *MdNup107a/b*, *MdNup50a/b*, *MdNup98a/b*, *MdNup136a/b, MdSec13a/b*, *MdSeh1a/b*, *MdNDC1a/b*, *MdRAE1a/b*), analyzing their expression levels separately was difficult. Therefore, we performed a combined analysis of the expression levels of each pair of homologous genes. The 38 candidate apple Nups produced varying expression patterns in different tissues. For example, *MdNup35, MdNup54, MdNup62, MdNup133, MdNup160, MdSeh1, MdCG1, MdRAE1, MdTPR, MdALADIN*, and *MdCPR5* were most highly expressed in the buds, implying they may be involved in apple flower bud induction. In contrast, *MdNup43* and *MdNup98* were highly expressed in fruits, whereas *MdNup107*, *MdGLE1*, and *MdNDC1* expression levels were high in the stems. Moreover, the highest *MdNup88*, *MdNup96*, *MdNup155*, *MdHOS1*, and *MdGP210* expression levels were detected in the roots.

### Apple Nups expression patterns in response to IAA treatments during the flower induction period

**Figure 6:**
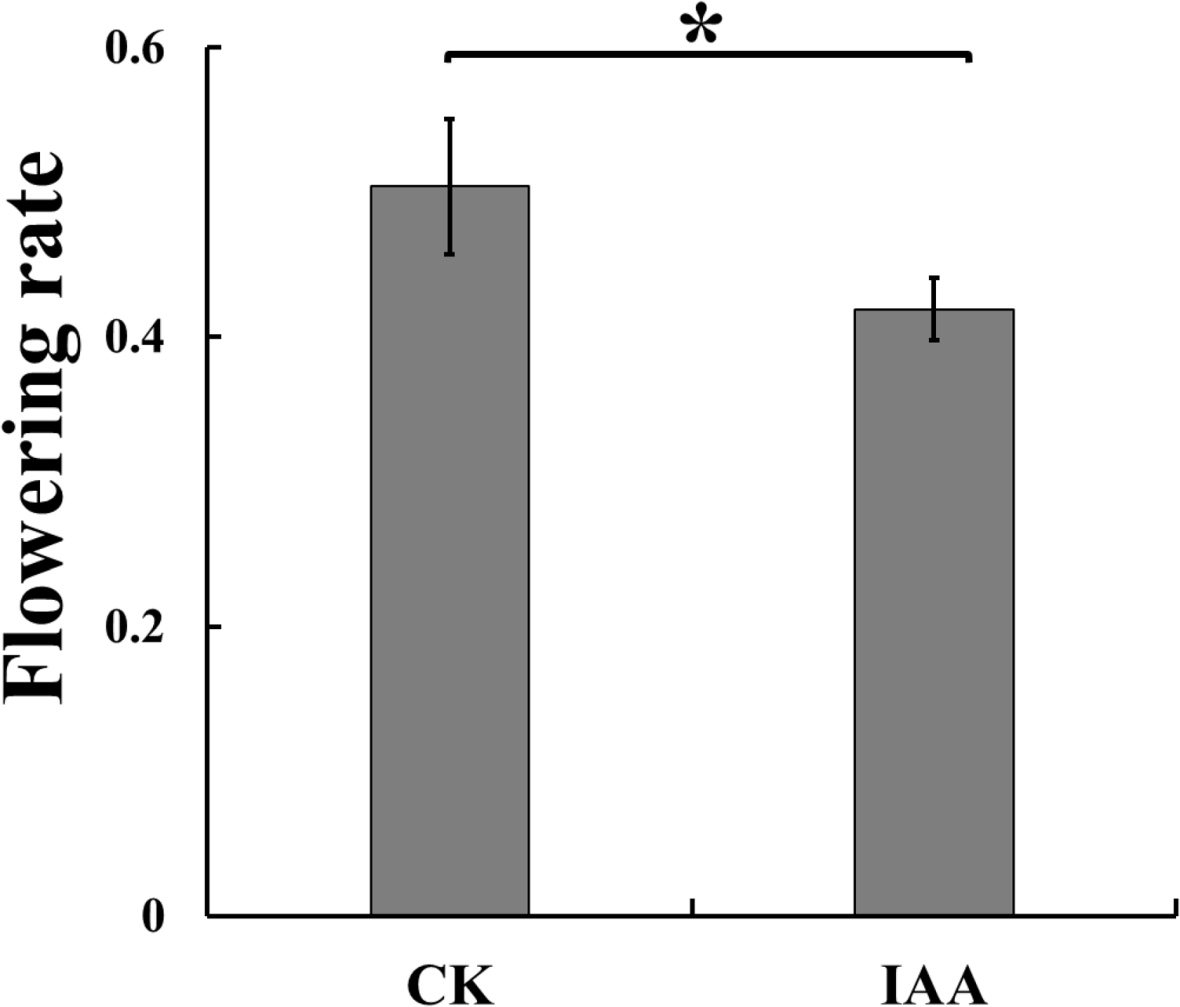
Flowering rate of ‘Fuji’ apple trees following control (CK) and IAA treatments. The presented data are derived from five biological replicates. The histograms were made by Excel 2007. Asterisks denote a significant difference as determined by the *t*-test: *P < 0.05.

**Figure 7:**
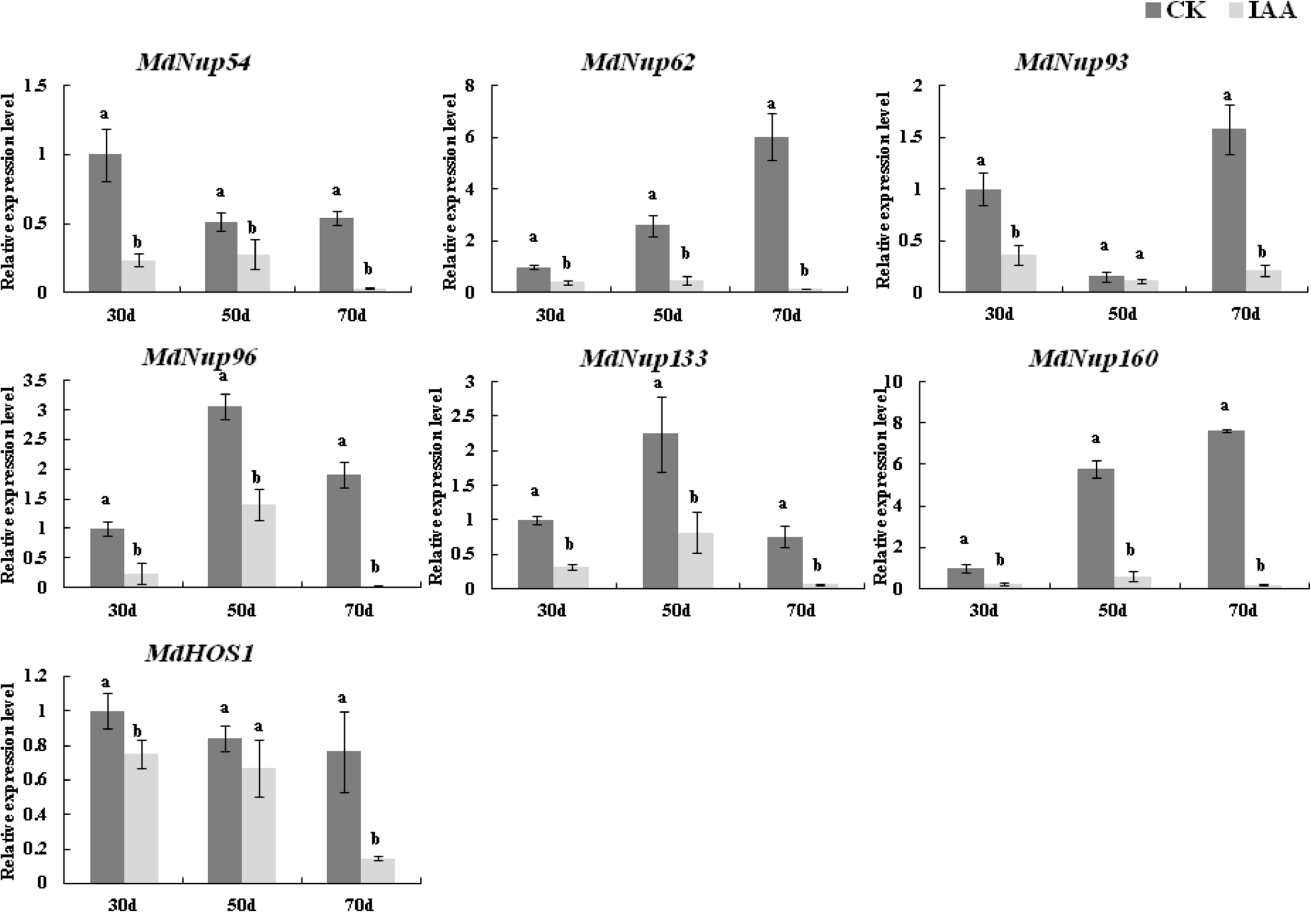
Expression patterns of seven candidate *MdNPCs* in apple buds treated with IAA. Control buds were treated with water. Samples were collected at 30, 50, and 70 days after full bloom (DAFB). Each sample was analyzed with three biological replicates, each comprising three technical replicates. The histograms were made by Excel 2007. Means followed by different lowercase letters are significantly different at the 0.05 level.

We investigated the effect of an IAA treatment on apple flower induction. The flowering rate following the IAA treatment was 41.9%, which was significantly lower than the 50.4% flowering rate after the water (control) treatment (Fig. 6). Previous studies have confirmed that some Nups (*AtNup54*, *AtNup62*, *AtNup96*, *AtNup160*, and *AtHOS1*) affect flowering time in *Arabidopsis thaliana*^1,23–25^. And the tissue-specificity analysis found that *MdNup54, MdNup62, MdNup133,* and *MdNup160* were most highly expressed in buds, and *MdNup*93 was also highly expressed in buds, suggesting that these genes may be involved in the apple flowering pathway. So we analyzed the transcription of these seven candidate *MdNups* . And the expression of all seven candidate genes was significantly inhibited by the IAA treatment (Fig. 7). Moreover, the transcription of *MdNup54, MdNup62, MdNup96, MdNup133, MdNup160* were significantly inhibited at 30, 50, and 70 days after flowering. In contrast, the *MdNup93* and *MdHOS1* expression levels were not significantly different following the IAA and water treatments at 50 days after flowering, but the expression levels were lower in the IAA-treated samples than in the control samples at 30 and 70 days after flowering. Accordingly, the IAA treatment can significantly inhibit the expression of these genes.

### *MdNup62* interacts with *MdNup54*

**Figure 8:**
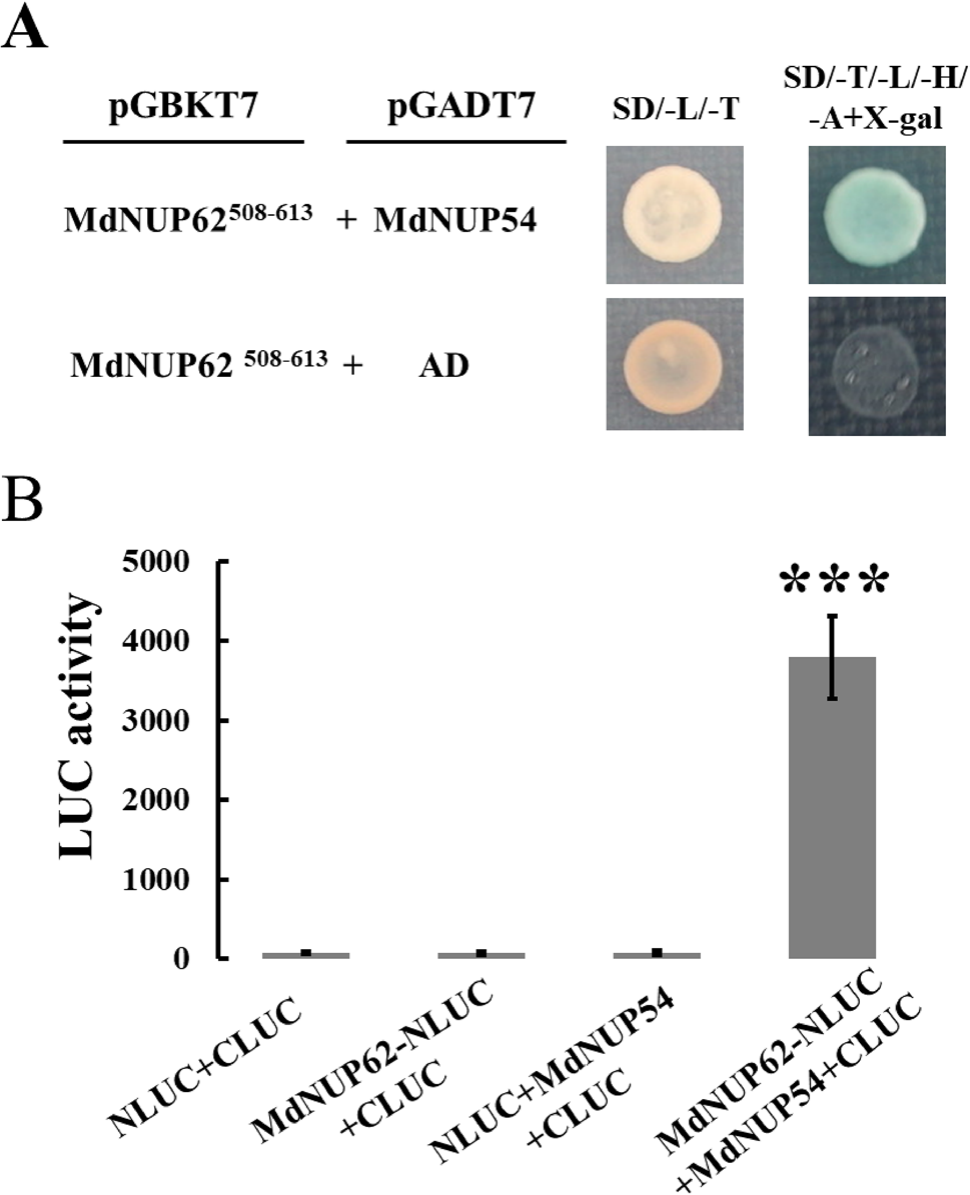
Interaction between MdNup62 and MdNup54. (**A**) MdNup62^508–613^ interacted with MdNup54 in Y2H assays. The *MdNup62*^508–613^ truncated sequence was cloned into pGBKT7, whereas *MdNup54* was cloned into pGADT7. Empty pGADT7 plus *MdNup62*^508–613^-pGBKT7 was used as a control. Yeast cells grown in SD/ − Trp/ − Leu medium and SD/ − Trp/ − Leu/ − His/ − Ade/ + X-α-gal medium are presented. (**B**) The luciferase complementation experiment involving tobacco leaves revealed the interaction between MdNup62 and MdNup54. Empty NLUC and empty CLUC, *MdNup62*-NLUC and empty CLUC, and empty NLUC + *MdNup54*-CLUC were used as controls. The luciferase complementation experiment was repeated three times, with consistent results. Asterisks denote significant differences as determined by the *t*-test: *P < 0.01.

Previous studies found that *Nup54*, *Nup58,* and *Nup62* form a complex and function together in metazoan, and the interaction between the other two members in *A. thaliana Nup62* subcomplex, *AtNup58* and *AtNup62*, were also reported^31^. And *MdNup62* and *MdNup54* form the *Nup62* subcomplex, we hypothesized that these two proteins interact with each other. To test this hypothesis, we performed a Y2H experiment. First, we observed that the truncated MdNup62^1–507^ was self-activating, but MdNup62^508–613^ was not (Fig. S2). Therefore, *MdNup62*^508–613^-pGBKT7 was selected as the bait and was included in a co-transformation of yeast cells along with *MdNup54*-pGADT7. And they could grow normally on SD/ − Trp/ − Leu medium and SD/ − Trp/ − Leu/ − His/ − Ade/ + X-α-gal medium, and bacame significantly blue on SD/ − Trp/ − Leu/ − His/ − Ade/ + X-α-gal medium. But the co-transformation of *MdNup62*^508–613^-pGBKT7 and empty- pGADT7 could only grow on the SD/ − Trp/ − Leu medium, and could neither grow nor turn blue on the SD/ − Trp/ − Leu/ − His/ − Ade/ + X-α-gal medium. So the Y2H assay confirmed that MdNup62 can interact with MdNup54 (Fig. 8). This interaction was further verified in a split-LUC complementation assay. The co-expression of *MdNup62*-NLUC and *MdNup54*-CLUC resulted in higher LUC activity than the other combinations (Fig. 8). These results confirmed the interaction between MdNup62 and MdNup54.

### *MdNup54* interacts with *MdKNAT4* and *MdKNAT6*

**Figure 9:**
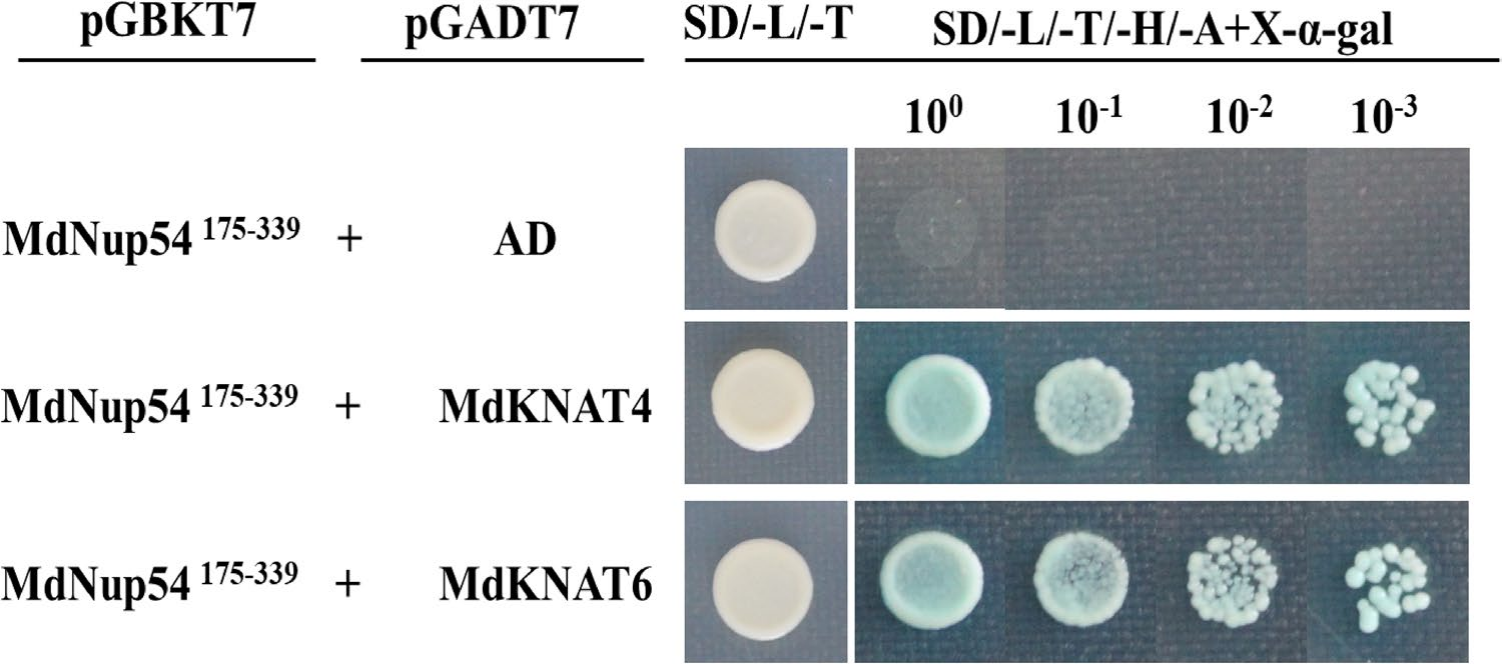
Interactions between MdNup54^175–339^ and MdKNAT4 as well as MdKNAT6 in Y2H assays. The *MdNup54*^175–339^ truncated sequence was cloned into pGBKT7, whereas *MdKNAT4* and *MdKNAT6* were cloned into separate pGADT7 vectors. Empty pGADT7 plus *MdNup54*^175–339^-pGBKT7 was used as a control. Additionally, 10^0^, 10^−1^, 10^−2^, and 10^−3^ represent the dilutions of the yeast solution. Yeast cells grown in SD/ − Trp/ − Leu medium and SD/ − Trp/ − Leu/ − His/ − Ade/ + X-α-gal medium are presented.

Although MdNup54 is an important component of the Nup62 subcomplex and influences plant growth and development, there has been relatively little research on the *MdNup54* gene in plants. Thus, we conducted a Y2H assay to explore the biological processes MdNup54 may contribute to. First, we observed that the truncated MdNup54^1–90^ was self-activating, but MdNup54^175–339^ was not (Fig. S3). Accordingly, *MdNup54*^175–339^-pGBKT7 was selected as the bait and inserted into yeast cells, which were then transformed with plasmids from the apple bud plasmid library to screen for interacting proteins. Both MdKNAT4 and MdKNAT6 were detected as potential interacting proteins. The *MdKNAT4* and *MdKNAT6* sequences were cloned and ligated to separate pGADT7 vectors. To conduct a Y2H assay, we co-transformed yeast cells with *MdKNAT4*-pGADT7 or *MdKNAT6*-pGADT7 and *MdNup54*^175–339^-pGBKT7. And these two kinds co-transformed yeast cells could both grow normally on SD/ − Trp/ − Leu medium and SD/ − Trp/ − Leu/ − His/ − Ade/ + X-α-gal medium, and became significantly blue on SD/ − Trp/ − Leu/ − His/ − Ade/ + X-α-gal medium. But the control could only grow on SD/ − Trp/ − Leu medium. The assay results verified that both MdKNAT4 and MdKNAT6 can interact with MdNup54 (Fig. 9).

## Discussion

The NPC controls the communication between the nucleus and cytoplasm, with consequences for diverse biological processes that influence plant growth and development. The relatively few studies that have examined plant NPC have been limited to model species, such as *A. thaliana*. Therefore, only the *A. thaliana* NPC has been systematically identified. We know very little about the corresponding apple genes. Thus, we identified and attempted to functionally characterize the apple NPC.

### Genome-wide identification and characterization of Nups in apple

**Figure 10:**
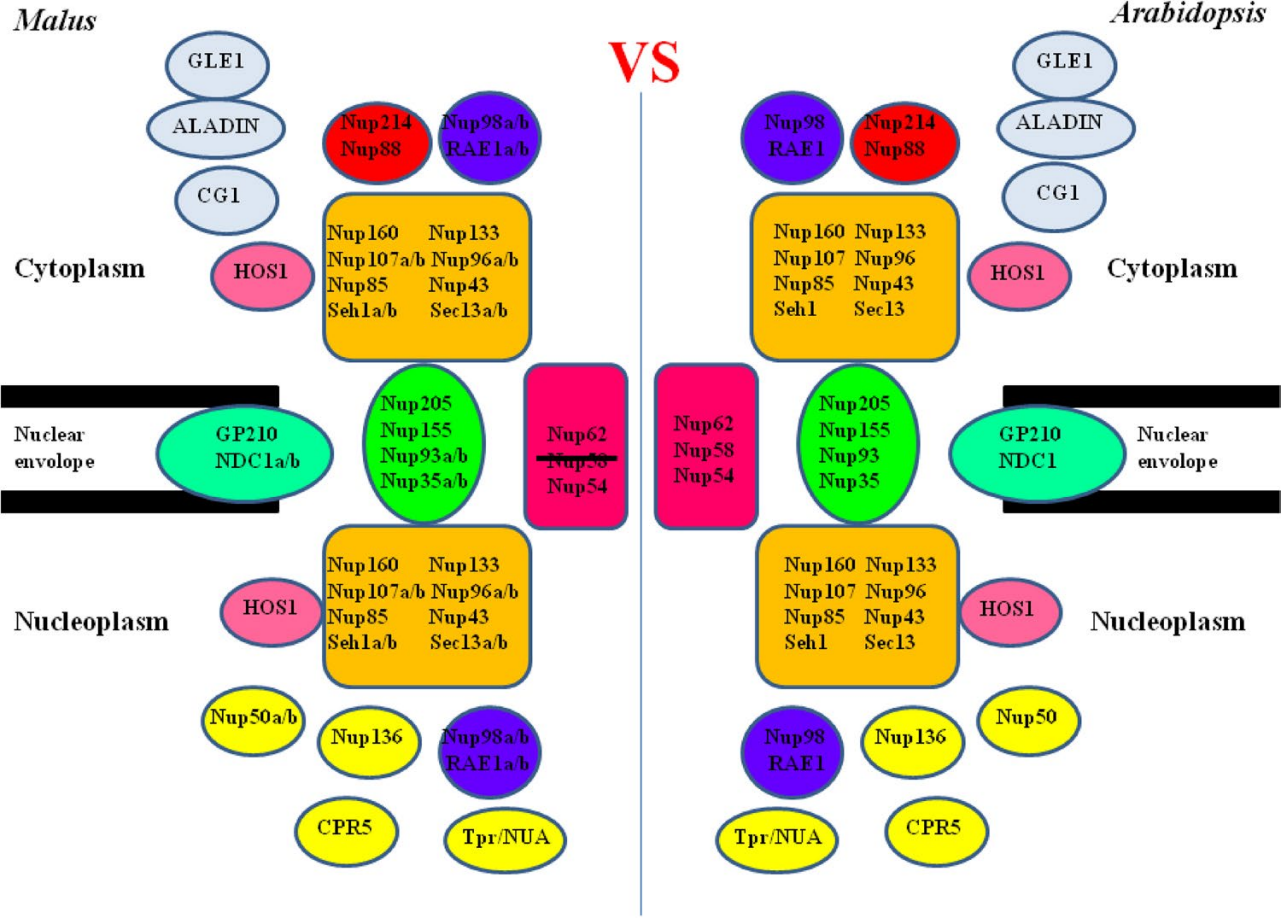
Comparison between the apple and *Arabidopsis thaliana* NPC. The positions of each Nups refer to previous studies^1,2,47^.

We identified 38 candidate apple Nups and more than 30 *A. thaliana* genes. Almost all of the *A. thaliana* Nups had a corresponding homologous sequence in apple, suggesting these are conserved plant genes. However, apple homologs of the *A. thaliana Nup58* gene was not detected (Fig. 10). *A. thaliana Nup58* belongs to *Nup62* subcomplex, which means that lacking *Nup58* of the apple *Nup62* subcomplex must be functionally different from that of *A. thaliana*. The lack of *Nup58* gene is bound to lead to differences in the function of the apple and *A. thaliana*. NPC. Additionally, because apple is a woody plant species, its genome is more complex than that of *A. thaliana*, with 11 genes (*Nup35*, *Nup50*, *Nup93*, *Nup96*, *Nup98*, *Nup107*,*Nup136, Sec13*, *Seh1*, *NDC1*, and *RAE1*) having two alleles each, whereas only *Nup98* and *Nup50* had two alleles in *A. thaliana*^2^. Although there are some differences between the apple and *A. thaliana* NPC, the basic structures of the encoded proteins are the same, including the *Nup62*, *Nup93*, and *Nup107–160* subcomplexes as well as other Nups inside and outside the nuclear pore^1,2^. These findings imply the apple and *A. thaliana* NPC are functionally similar.

The details regarding the introns and exons of the apple Nups suggested there is a lack of similar structures among the genes, including between the genes within the same subcomplex, further demonstrating the relative functional independence of the apple Nups. Apple Nups encode a class of compounds with no conserved domain among all members, unlike the members of other apple gene families (e.g., *IDD*, *GRF*, *GASA* and *SBP-box* genes)^27,28,32,33^. Only some of the apple Nups encode a conserved domain. Therefore, these proteins may have similar functions. Conserved domains were not detected among the other apple Nups, implying a lack of functional redundancy.

We also analyzed the phylogenetic relationships between Nups. First, we constructed a phylogenetic tree based on the *A. thaliana* and apple Nups and divided 69 Nups into three groups, consisting of 20, 30, and 19 members (Fig. 3). The apple Nups are most closely related to the corresponding *A. thaliana* Nups (e.g., *MdNup96a/b* and *MdCG1* are most closely related to *AtNup96* and *AtCG1*, respectively). These results suggest Nups are conserved and may have similar functions in diverse species.

The *Nup62* subcomplex occupies an important position in the nuclear pore (i.e., central pore channel). An analysis of the evolutionary relationships involving *MdNup54* and *MdNup62* indicated that both genes are closely related to genes in other Rosaceae species, especially to genes in *Pyrus communis*, which is in the same subfamily as apple.

### *Apple *Nups expression patterns

We performed a qRT-PCR assay to study the expression levels of apple Nups in six tissues of ‘Nagafu No. 2’. The *MdNup35*, *MdNup54*, *MdNup62*, *MdNup133*, *MdNup160*, *MdSeh1*, *MdCG1*, *MdRAE1*, *MdTPR*, *MdALADIN*, and *MdCPR5* showed the highest expression in the buds, suggesting that they may be involved in the apple flowering pathway. NPC acts as a barrier and regulats the flow of RNA and proteins into and out of the nucleus. Therefore, the level of their expression should have an important effect on the growth and development of apple flower buds. Previous *A. thaliana* studies confirmed that the deletion of *AtNup54*, *AtNup62*, *AtNup160,* and *AtTPR* results in early flowering^1^. And this supports our speculation very well. Both *MdNup43* and *MdNup98* were highly expressed in fruits, implying they may contribute to apple fruit development. In contrast, *MdNup107*, *MdGLE1*, and *MdNDC1* expression levels were highest in the stems, suggesting they are important for apple stem growth and development. Additionally, *MdNup88*, *MdNup96*, *MdNup155*, *MdHOS1*, and *MdGP210* expression levels were highest in the roots, indicative of their potential roles in root development. However, Tissue specific expression only provides reference for the potential function of Nups, which need to be experimentally verified.

*Arabidopsis thaliana* studies have confirmed that some Nups (*AtNup54*, *AtNup62*, *AtNup96*, *AtNup160*, and *AtHOS1*) affect flowering time^1,23–25^. However, little is known about their potential roles in apple-induced flowering. Therefore, we investigated the expression patterns of *MdNup54*, *MdNup62*, *MdNup93*, *MdNup96*, *MdNup133*, *MdNup160*, and *MdHOS1* to preliminarily explore whether they are associated with IAA-mediated flowering. We determined the flowering rates of ‘Nagafu No. 2’ treated with IAA. Our data indicated that the IAA treatment significantly inhibited flowering, which was consistent with the results of previous studies on apple and other species^34,35^. We subsequently performed a qRT-PCR assay to quantify the expression of these genes. The expression levels of the IAA-treated plants were significantly lower than those of the controls, suggesting these seven apple Nups are responsive to the application of exogenous IAA. Earlier investigations revealed that *A. thaliana Nup62, Nup96*, and *Nup160* genes are involved in the auxin signaling pathway^36^, which is consistent with the results of this study. However, we cannot determine whether apple Nups are involved in the IAA treatment resulting in the reduction of flowering rates. And we only speculate that apple Nups are involved in the IAA regulation of apple flowering pathway. But it is certain that IAA treatment will reduce the expression of apple Nups and flower rates. And the relationship between apple Nups and flower rates needs to be verified by follow-up experiments.

### Preliminary functional characterization of the *Nup62* subcomplex in apple

The apple *Nup62* subcomplex has only two members (*MdNup54* and *MdNup62*), whereas the corresponding complex in *A. thaliana* has three members (*AtNup54*, *AtNup58,* and *AtNup62*)^1,2^. And we confirmed that *MdNup54* and *MdNup62* interact in apple. In other words, the complete biological functions of *MdNup54* and *MdNup62* may require the interaction between these two proteins. Additionally, because of a lack of *Nup58*, the apple *Nup62* subcomplex and the corresponding *A. thaliana* subcomplex may be functionally diverse.

We conducted a Y2H assay to verify the interactions between *MdNup54* and two members of the *KNOX* family (*MdKNAT4* and *MdKNAT6*). To the best of our knowledge, this is the first study to reveal an interaction between an NPC and members of the *KNOX* family. The *KNOX* family members have crucial functions related to plant hormone signaling^37–39^, as well as leaf^40,41^ and flower development^42^. The apple *MdKNAT4* and *MdKNAT6* genes are homologs of the *A. thaliana AtKNAT4* and *AtKNAT6* genes, respectively. Earlier studies proved that *AtKNAT4* influences seed dormancy^43^, while *AtKNAT6* plays an important role in maintaining meristem integrity and flowering^42^. Apple MdNup54 may also affect similar pathways. Notably, the *KNOX* family is involved in cytokinin and gibberellin signaling pathways^37–39^. These two hormones are closely related to apple flowering^27,44,45^, suggesting that apple *MdNup54* may indirectly affect cytokinin and gibberellin signaling pathways by controlling the transport of *KNOX* genes into the nucleus, thereby regulating apple flowering. But previous studies have shown that selective transport between the nucleus and cytoplasm depends on nuclear transport receptors (importin and exportin), which bind cargos and interact with the NPC selective barriers for cargo transport^3,4,46^. Recently, researchers have found that *Nup85* and *MED18* can interact directly with each other. And the two mutants had the same abiotic stress phenotype^22^. Therefore, the relationship between *Nup54* and *KNOX* genes may provide a hypothesis for NPC studies that Nups may interact directly with transcription factors to control their nuclear transport.

## Supplementary information

Supplementary information 1

Supplementary information 2
