# Supplementary material for: Genomic identification and expression analysis of nuclear pore proteins in Malus domestica
Source: Sci Rep. 2020 Oct 15;10:17426. doi: 10.1038/s41598-020-74171-0 (PMC7566457; doi:10.1038/s41598-020-74171-0)
Supplement: Supplementary file 1 — Supplementary information 1 [file 41598_2020_74171_MOESM1_ESM.docx]

# Genomic Identification and Expression Analysis of Nuclear Pore Proteins in Malus domestica

Chenguang Zhang^1^**^†^**, Na An^1^**^†^**, Peng Jia^1^**^†^**, Wei Zhang^1^, Jiayan Liang^1^, Xu Zhang^1^, Hua Zhou^1^, Wenchun Ma^1^, Mingyu Han^1^*, Libo Xing^1^* and Xiaolin Ren^1^*

^1^College of Horticulture, Northwest A&F University, Yangling, China

**^†^**Equal contributors

*Correspondence:

Mingyu Han: hanmy@nwsuaf.edu.cn

Libo Xing: libo_xing@nwsuaf.edu.cn

Xiaolin Ren: [renxl@nwsuaf.edu.cn](mailto:renxl@nwsuaf.edu.cn)


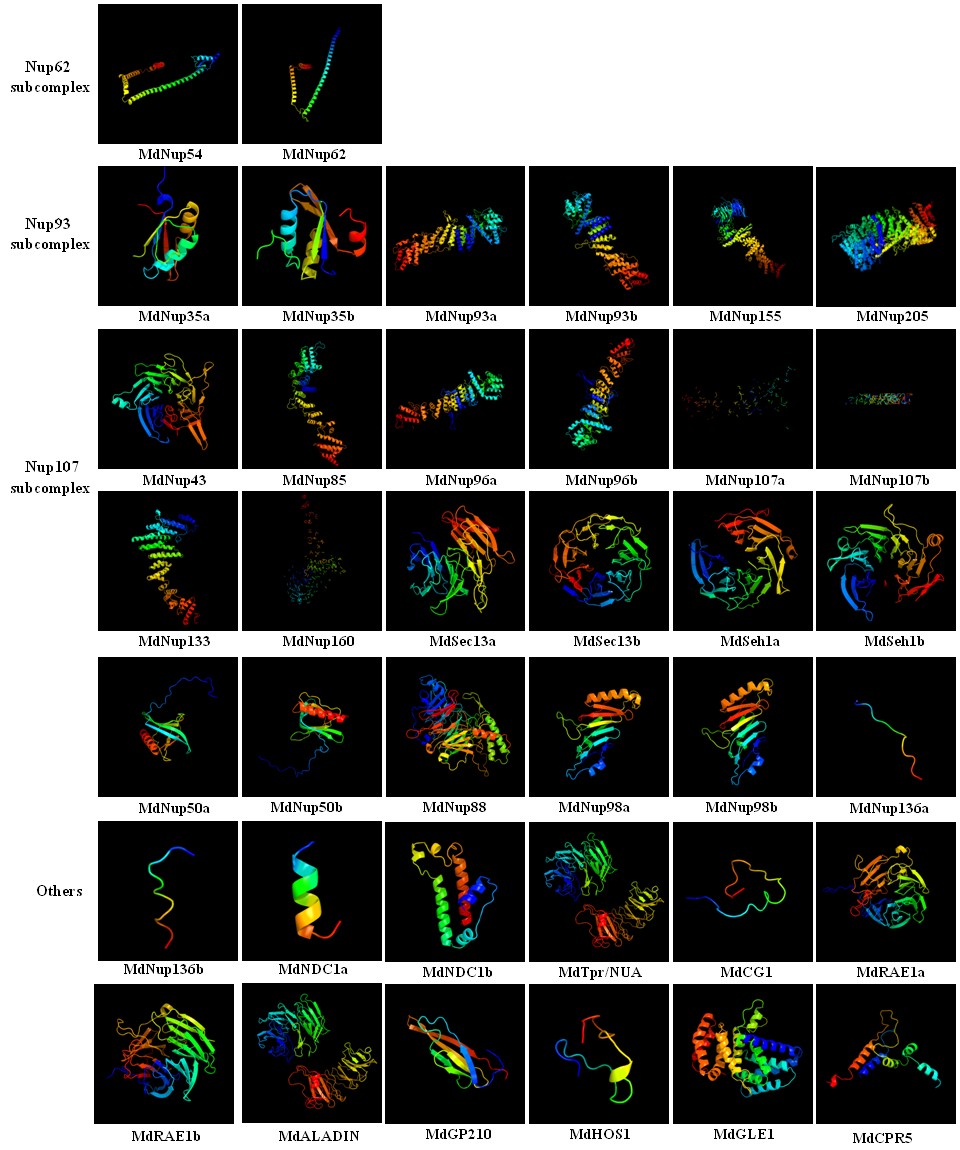


**Figure S1**


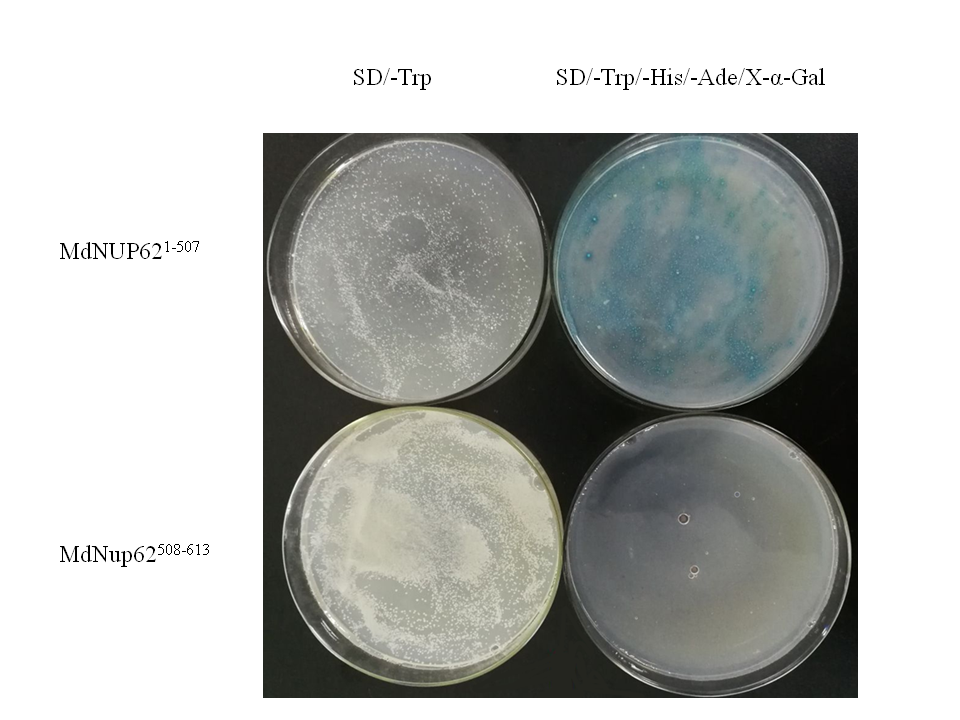


**Figure S2**


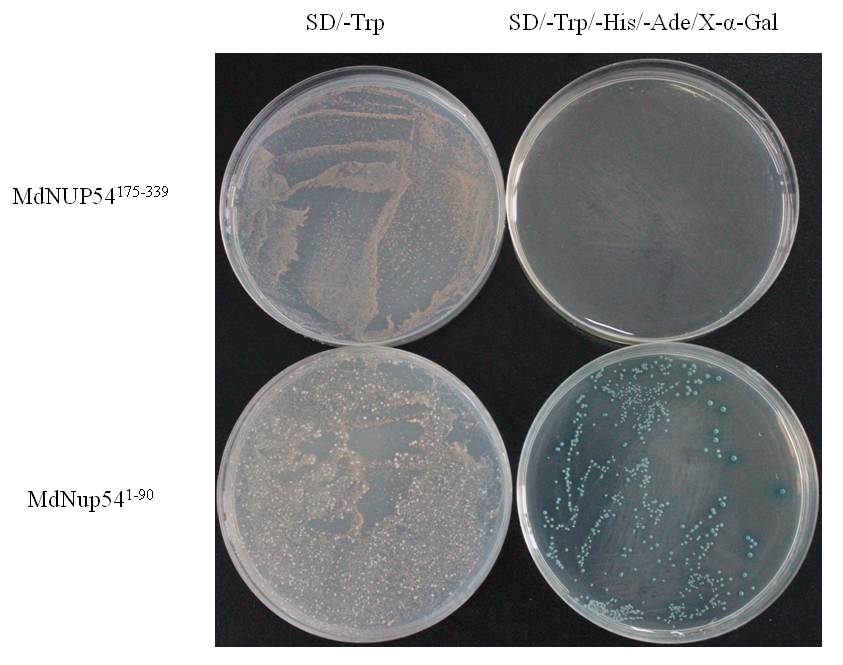


**Figure S3**

**Table S1** qRT-PCR primers for *MdNPCs*

| Gene name | Gene ID | Primer sequences |
| --- | --- | --- |
| NUP35-F | MD09G1205800 | ACCTTCCACCACCTCCGCTTTA |
| NUP35-R | MD09G1205800 | GCATACGAAGTGCTTGCCTCTG |
| NUP43-F | MD10G1281400 | TCCGAACCCAGCAACCCTAACC |
| NUP43-R | MD10G1281400 | CACACTCCGACCCACCATCCAT |
| NUP50-F | MD09G1214400 | TGTTAAGGTTCGTCGCCAGCAG |
| NUP50-R | MD09G1214400 | AACTCGTGTCTCACAGGCAGGT |
| NUP54-F | MD16G1117500 | AGAGGGCAAGGGTTTCCGACTG |
| NUP54-R | MD16G1117500 | TCCGTCTGCTGCTGTAACACCT |
| NUP62-F | MD07G1110700 | GGCTTCCTCCGCTTCACAATCC |
| NUP62-R | MD07G1110700 | CGGTGCTAGGAGCCTGAGAAGT |
| NUP85-F | MD15G1093200 | GGCTCCAATGACACCGCTATGG |
| NUP85-R | MD15G1093200 | CGCCAGTTCGCATCGCCATT |
| NUP88-F | MD01G1152200 | AGAGACGAAGTGGAGTGGGTCC |
| NUP88-R | MD01G1152200 | GCGAAGAAGCGATGACGGAAGT |
| NUP93-F | MD12G1080600 | TTCTGAAGGTGCTGGTGCTGGA |
| NUP93-R | MD12G1080600 | TGCTGGTCCACGTTGCCTCT |
| NUP96-F | MD15G1399200 | GCATCAGCGTGCAGTGTTGGA |
| NUP96-R | MD15G1399200 | GGCTTGGCGTTGTGATTCTTGT |
| NUP98-F | MD14G1142000 | TGGACAGTTGCCTGCCACTCA |
| NUP98-R | MD14G1142000 | ACCTGACCCGCCTTTGTGACA |
| NUP107-F | MD09G1178100 | TGAGGTGGTGCTTCGCTGCT |
| NUP107-R | MD09G1178100 | TGCTGGGCTCTTCAAGGAACCT |
| NUP133-F | MD17G1113600 | TCCGTCAGACCCACACAAACCT |
| NUP133-R | MD17G1113600 | GTCTCAGCGGCAACACTTCCTC |
| NUP136-F | MD02G1257800 | CAACAGAACCTAACCAACTC |
| NUP136-R | MD02G1257800 | TATTCTTCGGTGAGACTTGT |
| NUP155-F | MD13G1020400 | TAGGTGGTGGTGACCGTGGTAC |
| NUP155-R | MD13G1020400 | CCGTGTGACATGGTGCTGTGAA |
| NUP160-F | MD10G1009800 | GACATTGGCAGGCATGGAGGTC |
| NUP160-R | MD10G1009800 | ATGGCGACGGAAGAGGCGATAT |
| NUP205-F | MD02G1032900 | GGAGCAAGCAGCCCAACTTCAT |
| NUP205-R | MD02G1032900 | CAGAGCAACCTGGCACAGCATA |
| ALADIN-F | MD12G1112000 | CGGACGATGTAGCCAAGGACAC |
| ALADIN-R | MD12G1112000 | GGCGGAAGAGAGGAGTGAGGTT |
| CG1-F | MD04G1000600 | TGAGGGCAGCAGCATACGATGA |
| CG1-R | MD04G1000600 | GGCTGGCAAGGGTAGAGTTTGG |
| GLE1-F | MD13G1104500 | TGCCGAGTTACACAGAGCTTGC |
| GLE1-R | MD13G1104500 | GCCATCCATGCCCAACCTTCTC |
| GP210-F | MD17G1026700 | TGAGCCACGATTCCAGCACATG |
| GP210-R | MD17G1026700 | CCACACGAGCAACGGAAGAGTT |
| HOS1-F | MD04G1060900 | ACAGACCTGCATCGGAGTGTGA |
| HOS1-R | MD04G1060900 | GGACTCTGCTCCTCTTCCTCGT |
| NDC1-F | MD05G1278400 | GCTCCTCTTCTCCTTCGCACTC |
| NDC1-R | MD05G1278400 | CAACGACGCCACCGAAACAAAC |
| RAE1-F | MD08G1221600 | AGGTGCGATGCTGGGAGATACA |
| RAE1-R | MD08G1221600 | GCATGGCAACGGTCACTGGTT |
| SEC13-F | MD17G1042300 | CGATGGCTCCTGGTGCTCTAGT |
| SEC13-R | MD17G1042300 | CCACTGTTCCGTCCTGTGAAGC |
| SEH1-F | MD14G1122800 | CGAGGCTGCTGGCATTGTGAA |
| SEH1-R | MD14G1122800 | GTTGAGCCGAACCGCCGTTAA |
| TPR-F | MD05G1240600 | TGCTGAGAAGCGAGCTTGTGAC |
| TPR-R | MD05G1240600 | GTGTGAGAGTGCGGGCATTGTT |
| CPR5-F | MD01G1017700 | ATTGGGAGGGAACGGTAA |
| CPR5-R | MD01G1017700 | GGATACAGTATGGCAACCTT |
| ACTIN-F | MD04G1127400 | CAACTCATCCGAACCTCAAACC |
| ACTIN-R | MD04G1127400 | CGCTGTCCGCCATCTTCTACT |

**Table S2** Primers for *MdNup54* and *MdNup62*

| Gene name | Gene ID | Primer sequences |
| --- | --- | --- |
| MdNup54-F | MD16G1117500 | ATGTTCGGAGCTCAATCTTCGTC |
| MdNup54-R | MD16G1117500 | TTAGCTCCCGTTCTGTGTTGTTTC |
| MdNup62-F | MD07G1110700 | CCTCTCAATCACATAGAGCAGCAG |
| MdNup62-R | MD07G1110700 | GGAATTGAAGGTGGTTTACAATTCC |
| MdNUP54^1-90^-pGBKT7-F | MD16G1117500 | ATGGCCATGGAGGCCGAATTCATGTTCGGAGCTCAATC |
| MdNUP54^1-90^-pGBKT7-R | MD16G1117500 | CCGCTGCAGGTCGACGGATCCAGTTTGAGAAGAGAACGG |
| MdNUP54^175-339^-pGBKT7-F | MD16G1117500 | ATGGCCATGGAGGCCGAATTCAAGCCTGCTGGTGTATCG |
| MdNUP54^175-339^-pGBKT7-R | MD16G1117500 | CCGCTGCAGGTCGACGGATCCGGATATGGTTAATAGG |
| MdNUP62^1-507^-pGBKT7-F | MD07G1110700 | ATGGCCATGGAGGCCGAATTCATGTCGGGATTTTCATC |
| MdNUP62^1-507^-pGBKT7-R | MD07G1110700 | CCGCTGCAGGTCGACGGATCCCATTTGAAGTTGTCCCAC |
| MdNUP62^508-613^-pGBKT7-F | MD07G1110700 | ATGGCCATGGAGGCCGAATTCGTCAACACTACAGTATC |
| MdNUP62^508-613^-pGBKT7-R | MD07G1110700 | CCGCTGCAGGTCGACGGATCCCTTGTATATACGCTCAG |
| MdNUP54-pGADT7-F | MD16G1117500 | GCCATGGAGGCCAGTGAATTCATGTTCGGAGCTCAATCTTC |
| MdNUP54-pGADT7-R | MD16G1117500 | CAGCTCGAGCTCGATGGATCCTTAGCTCCCGTTCTGTGTTG |
| MdNUP54-CLUC-F | MD16G1117500 | GCTCTAGAATGTTCGGAGCTCAATCTTCG |
| MdNUP54-CLUC-R | MD16G1117500 | CGCGGATCCGCTCCCGTTCTGTGTTGTT |
| MdNUP62-NLUC-F | MD07G1110700 | GCTCTAGAATGTCGGGATTTTCATCCGG |
| MdNUP62-NLUC-R | MD07G1110700 | CGCGGATCCCGACATCCAGTATTTTGGAGC |
| MdKNAT4-F | MD02G1012900 | ATGGCGTTTCATCACCAGCAG |
| MdKNAT4-R | MD02G1012900 | CTACCTCTTGCGTTTGCTCTTGAG |
| MdKNAT6-F | MD13G1095800 | GATGGAGGAAATGTACGGATTGC |
| MdKNAT6-R | MD13G1095800 | GAGAGAATATACATGCATGCACCTC |
| MdKNAT4-pGADT7-F | MD02G1012900 | GCCATGGAGGCCAGTGAATTCATGGCGTTTCATCACCAG |
| MdKNAT4-pGADT7-R | MD02G1012900 | CAGCTCGAGCTCGATGGATCCCTACCTCTTGCGTTTGCTC |
| MdKNAT6-pGADT7-F | MD13G1095800 | GCCATGGAGGCCAGTGAATTCATGGAGGAAATGTACGGA |
| MdKNAT6-pGADT7-R | MD13G1095800 | CAGCTCGAGCTCGATGGATCTCATTCATTTGTAAAGAATG |
