# Supplementary material for: Genomic identification and expression analysis of nuclear pore proteins in Malus domestica
Source: Sci Rep. 2020 Oct 15;10:17426. doi: 10.1038/s41598-020-74171-0 (PMC7566457; doi:10.1038/s41598-020-74171-0)
Supplement: Supplementary file 2 — Supplementary information 2 [file 41598_2020_74171_MOESM2_ESM.docx]

Supplementary material

**Figure S1.** Diagram of the tertiary structures of apple NPC proteins. The predicted Apple NPC tertiary structures were analyzed with the PHYRE server (version 2.0) (http://www.sbg.bio.ic.ac.uk/phyre2/html/page.cgi?id=index).

**Figure S2.** The truncated MdNup62^508-613^ was not self-activated in Y2H assays. The indicated *MdNup54* regions were cloned into pGBKT7 (*MdNup54*^1–507^-pGBKT7 and *MdNup54*^508–613^-pGBKT7). Yeast cells grown in SD/−Trp medium and SD/−Trp/−His/−Ade medium are presented

**Figure S3.** The truncated MdNup54^175–339^ was not self-activated in Y2H assays. The indicated *MdNup54* regions were cloned into pGBKT7 (*MdNup54*^1-90^-pGBKT7 and *MdNup54*^175-339^-pGBKT7). Yeast cells grown in SD/−Trp medium and SD/−Trp/−His/−Ade medium are presented

**Table S1** Details regarding the qRT-PCR primers for apple NPCs

**Table S2** Details regarding the primers for *MdNup54* and *MdNup62*
